# Supplementary figures and images for: Sexual dimorphism in the tardigrade Paramacrobiotus metropolitanus transcriptome
Source: Zoological Lett. 2024 Jun 20;10:11. doi: 10.1186/s40851-024-00233-0 (PMC11191345; doi:10.1186/s40851-024-00233-0)

## Revigo TreeMap

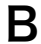

## Revigo TreeMap

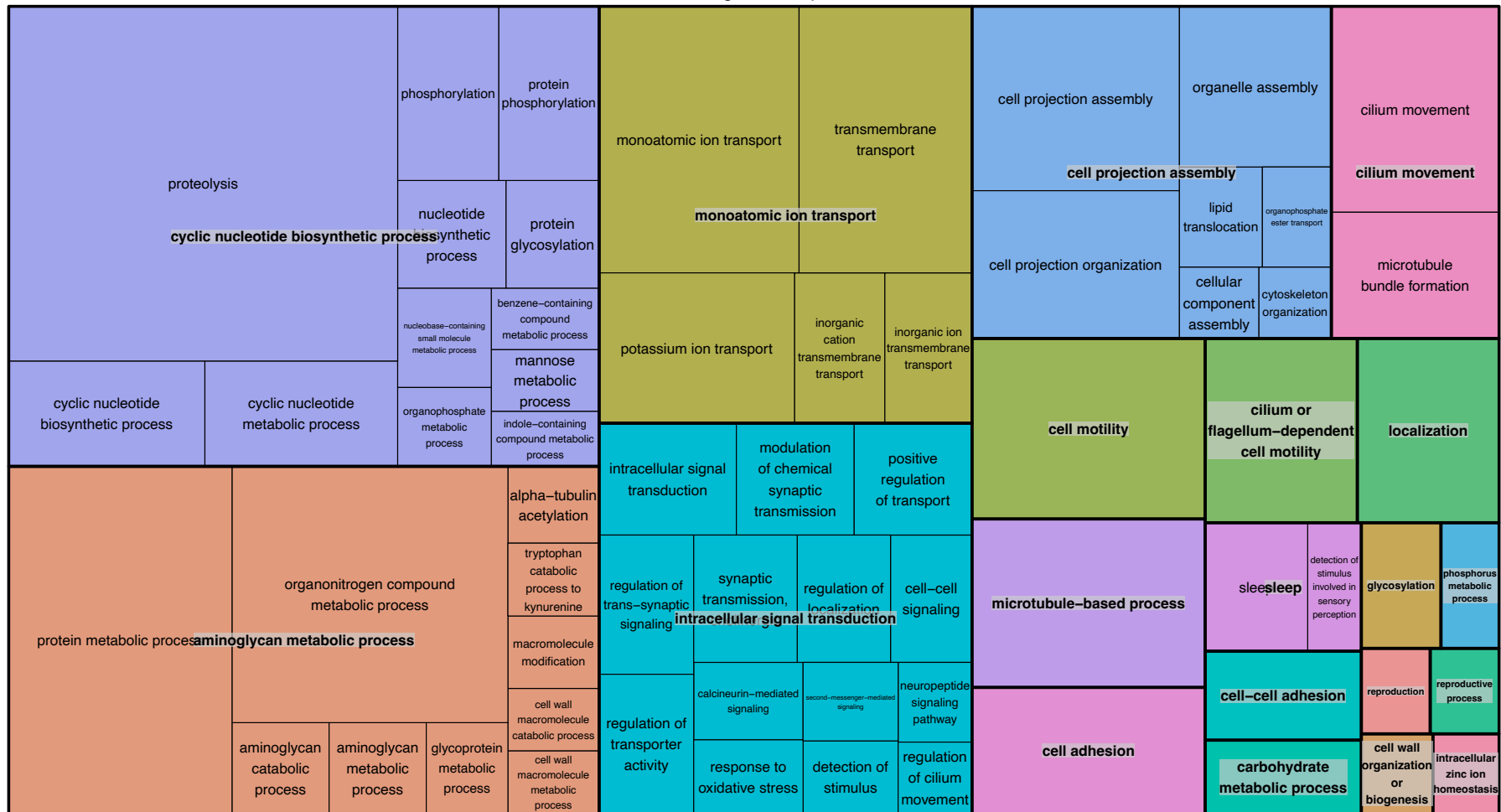

Supplement: Supplementary file 3 — Additional file 3: Figure S3. Expression of DMRT orthologs in H. exemplaris and R. varieornatus. Error bars indicate the standard deviation. On the X-axis, E and B time points indicate #day after oviposition (embryo) and #days after hatching (baby), and adults (active and tun). [file 40851_2024_233_MOESM3_ESM.pdf]

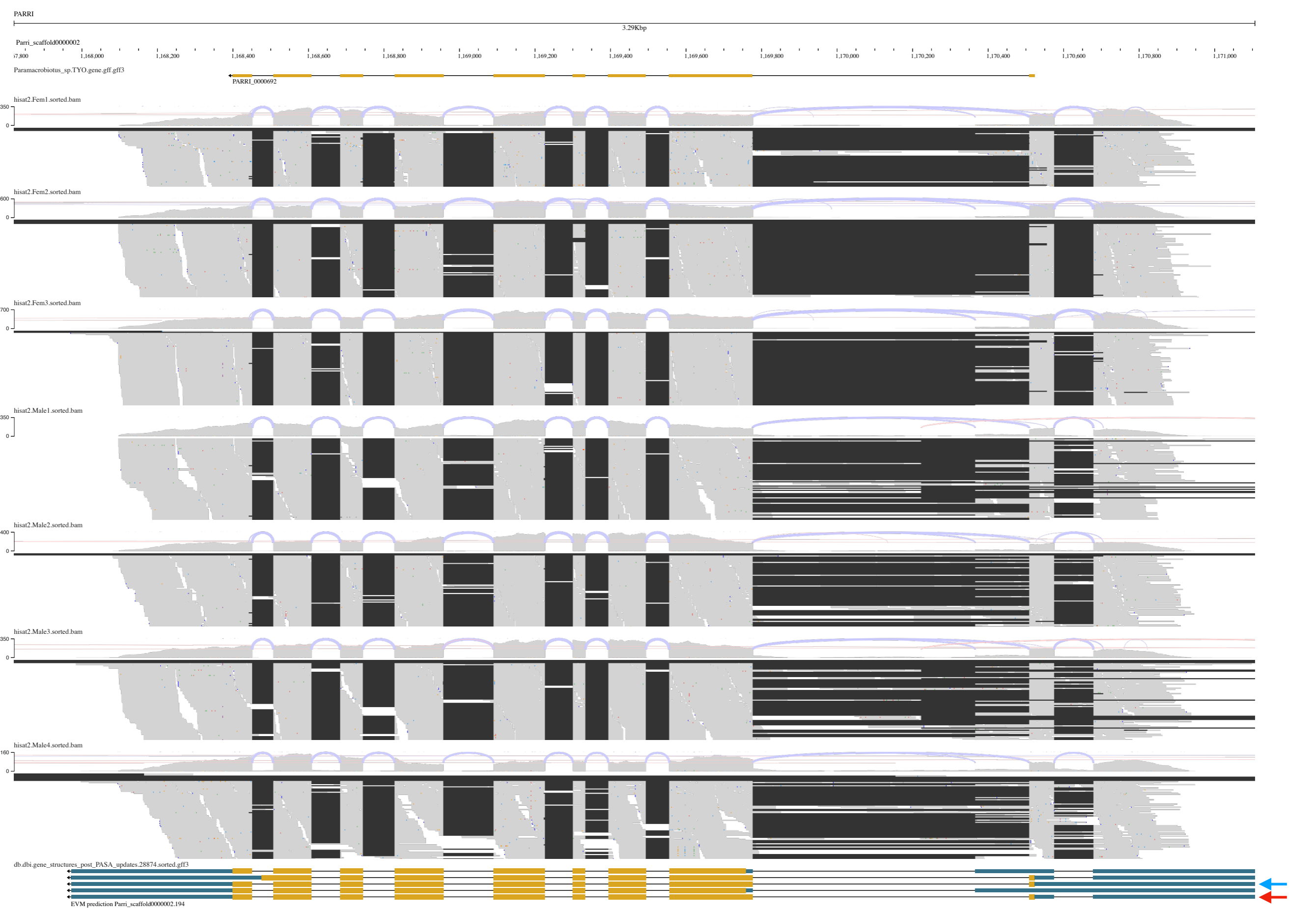

Supplement: Supplementary file 4 — Additional file 4: Figure S4. Structures of DMRT orthologs. [AB] Multiple alignment of the P. metropolitanus 3090/3093 complex orthologs [A] Amino acid sequences aligned by ClustalO and visualized by Seaview [B] Nucleotide sequences for the whole gene sequence (exon+intron) aligned by LAST through the MAFFT web site (https://mafft.cbrc.jp/alignment/server/index.html). The red line indicates the matching regions between the two sequences. [CDE] AlphaFold2 predicted the 3D structure of [C] full-length [D] DM domain, and [E] the CUE-DMA domain. The arrowheads in cyan and magenta indicate the DM and CUE-DMA domains, respectively. Dm indicates D. melanogaster. [file 40851_2024_233_MOESM4_ESM.pdf]

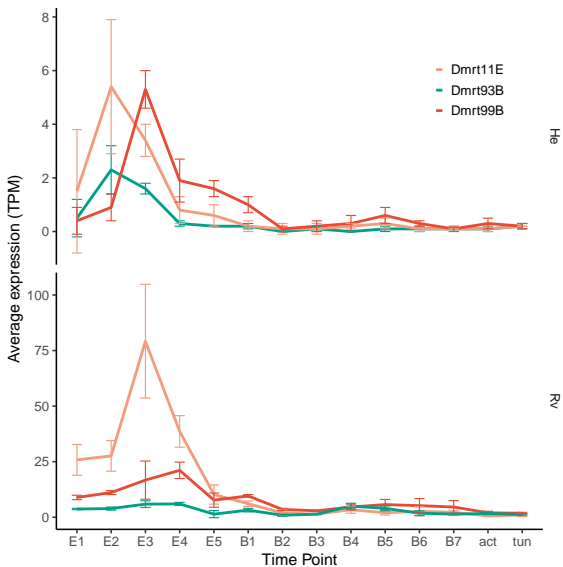

Supplement: Supplementary file 5 — Additional file 5: Table S1. Dmrt gene accession IDs and sequences used in the phylogenetic analysis. [file 40851_2024_233_MOESM5_ESM.pdf]

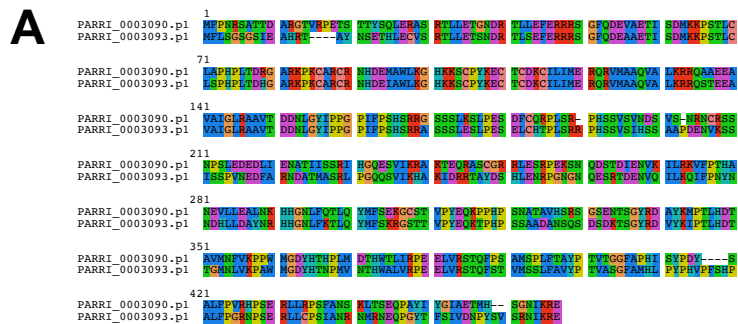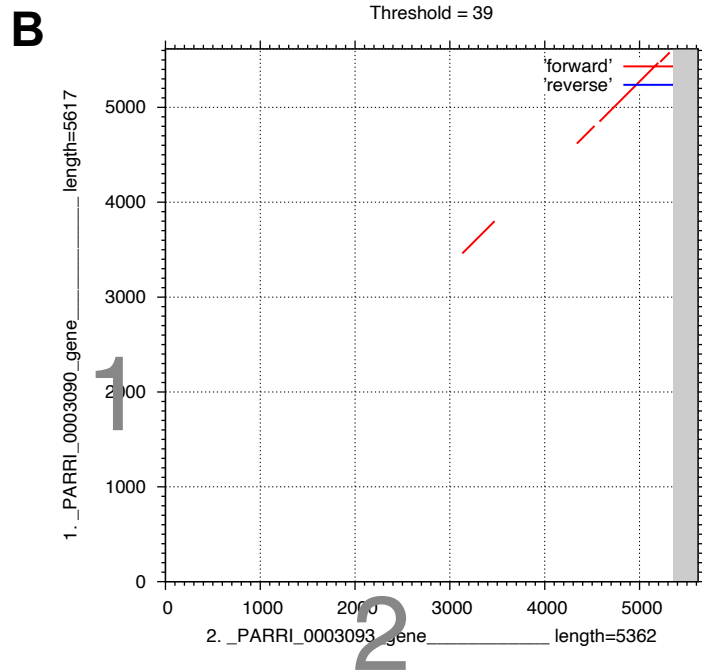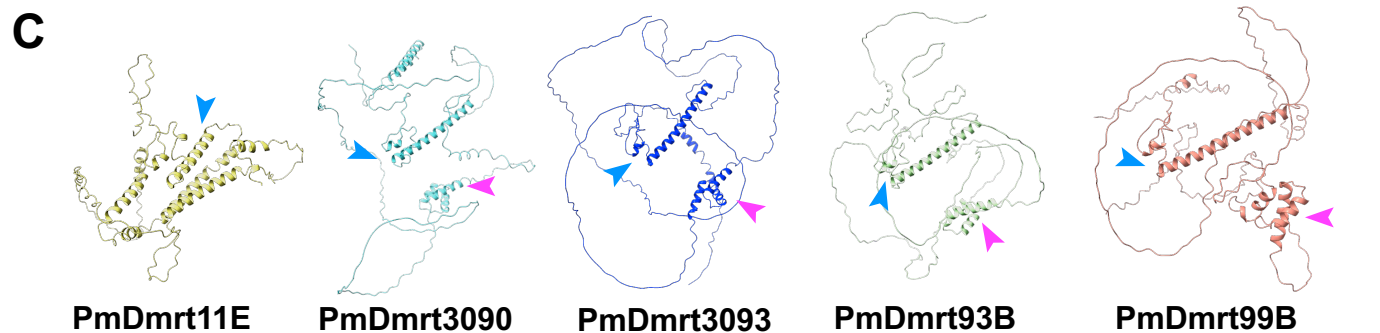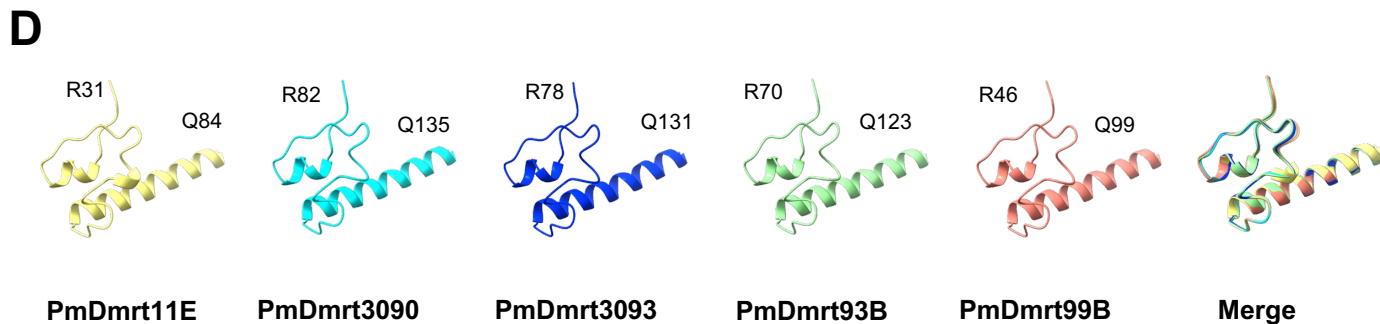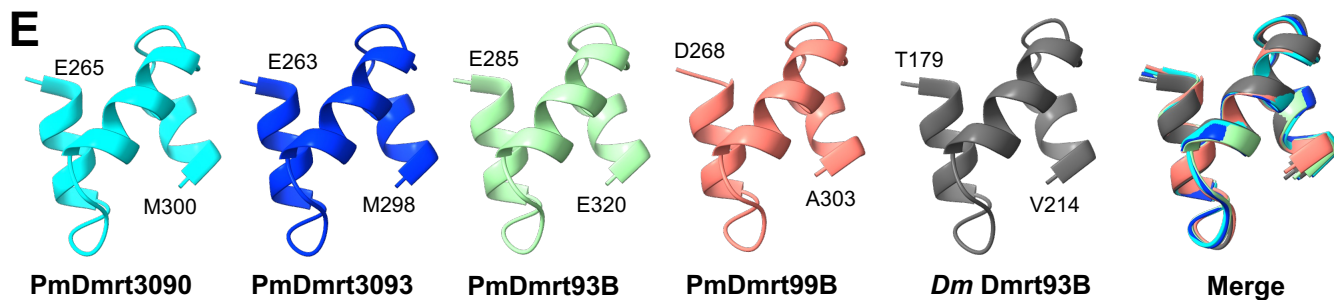

Supplement: Supplementary file 6 — Additional file 6: Table S2. Primer sequences for genotyping. [file 40851_2024_233_MOESM6_ESM.pdf]
